# Humeral head deviation and velocity in multidirectional instability of the glenohumeral joint: a cine magnetic resonance imaging study

**DOI:** 10.1016/j.jseint.2025.101419

**Published:** 2025-12-02

**Authors:** Kazuhisa Matsui, Takashi Tachibana, Katsuya Nobuhara, Yasushi Uchiyama

**Affiliations:** a Department of Physical Therapy, Faculty of Medical Science, Nagoya Aoi University, Nagoya-shi, Aichi-ken, Japan; b Department of Physiotherapy, Nobuhara Hospital, Tatsuno-shi, Hyogo-ken, Japan; c Department of Orthopaedic Surgery, Nobuhara Hospital and Institute of Biomechanics, Tatsuno-shi, Hyogo-ken, Japan; d Graduate School of Medicine, School of Health Sciences, Nagoya University, Nagoya-shi, Aichi-ken, Japan

**Keywords:** Dynamic instability, Glenohumeral joint, Humeral head deviation, Deviation velocity, Active shoulder rotation, Shoulder biomechanics

## Abstract

**Background:**

Manual clinical tests for shoulder instability rely heavily on subjective assessments of humeral head translation, making objective quantification challenging. This study hypothesized that patients with multidirectional glenohumeral instability (MDI) would demonstrate greater humeral head deviation and faster deviation velocity than healthy controls during active shoulder rotation, as assessed using cine magnetic resonance imaging (MRI).

**Methods:**

Fourteen participants (eight shoulders with MDI and 20 healthy shoulders) underwent cine MRI while performing active shoulder rotation with the arm at the side. Humeral head deviation, deviation amplitude, and deviation velocity were calculated and compared between the groups using Welch's *t*-test.

**Results:**

The MDI group showed significantly greater humeral head deviation, wider amplitude of deviation, and faster deviation velocity than the control group (*P* = .008 for anterior deviation, *P* = .009 for posterior deviation). The deviation amplitude exceeded 35% of the glenoid width in MDI shoulders, surpassing established clinical thresholds.

**Conclusion:**

Patients with MDI demonstrated quantifiable dynamic instability on cine MRI. This modality may provide objective support for clinical findings. However, validation in larger cohorts is warranted to confirm these findings, given the limited number of MDI shoulders (n = 8).

Multidirectional instability (MDI) of the glenohumeral joint is characterized by capsuloligamentous laxity and instability in both the anterior and posterior directions.^6^ This structural insufficiency impairs proprioception and alters rotator cuff muscle activation,^2^^,^^9^ contributing to delayed or asynchronous contraction timing.^4^^,^^10^^,^^12^ Consequently, dynamic stabilizers, particularly the rotator cuff muscles, play a crucial role in maintaining the centering of the humeral head within the glenoid fossa.^16^

Given that conservative management through neuromuscular rehabilitation is the first-line treatment for MDI,^2^^,^^6^ an accurate evaluation of dynamic stabilization mechanisms is crucial for treatment planning and monitoring.

Dynamic instability is commonly assessed through manual orthopedic examinations, such as the dynamic rotary stability test, wherein clinicians palpate the humeral head to detect abnormal translation during active shoulder rotation.^7^ However, these assessments are inherently subjective and depend on examiner experience, limiting their standardization and accuracy. This variability poses particular challenges for less experienced clinicians.

Although previous biomechanical studies have demonstrated that rotator cuff dysfunction can disturb the centering of the humeral head,^14^^,^^15^ most clinical evaluations have focused on static instability using tests such as the anterior and posterior drawer tests.^13^ Therefore, the intra-articular features of dynamic instability during active shoulder rotation remain poorly understood and are not readily detectable using current examination techniques.

To bridge this gap, dynamic imaging tools capable of capturing real-time intra-articular motion are required. Cine magnetic resonance imaging (cine MRI) offers a noninvasive approach for visualizing and quantifying humeral head translation during active shoulder rotation. Unlike ultrasound, which may restrict natural intra-articular motion of glenohumeral joint via compressing soft tissues by a probe, or radiography, which involves ionizing radiation, cine MRI allows assessment without interfering with joint mechanics.

Therefore, this study aimed to quantify dynamic glenohumeral instability in patients with MDI by measuring humeral head deviation and deviation velocity during active shoulder rotation using cine MRI. We hypothesized that patients with MDI would demonstrate (1) greater maximum deviation, (2) wider deviation amplitude, and (3) faster deviation velocity than healthy controls during active shoulder rotation.

## Materials and methods

### Participants

Fourteen participants were enrolled in this study, including four patients with bilateral multidirectional instability (MDI; eight shoulders) and 10 healthy adults (20 shoulders). The MDI group consisted of two women and two men (mean age, 29.8 ± 8.4 years), and the control group included two women and eight men (mean age, 27.9 ± 6.2 years).

MDI was diagnosed by orthopedic surgeons based on clinical criteria, including symptomatic instability (eg, discomfort, pain, or apprehension), a positive sulcus sign, and at least two additional positive findings among the anterior drawer, posterior drawer, load-and-shift, or apprehension tests.^1^ Control group participants had no history of cervical, thoracic, or upper extremity injury and no reported neurological symptoms.

The exclusion criteria for both groups included a history of shoulder surgery, prior conservative treatment for shoulder instability, pain precluding testing, and any contraindications to MRI.^3^

### Procedures

**Figure 1:**
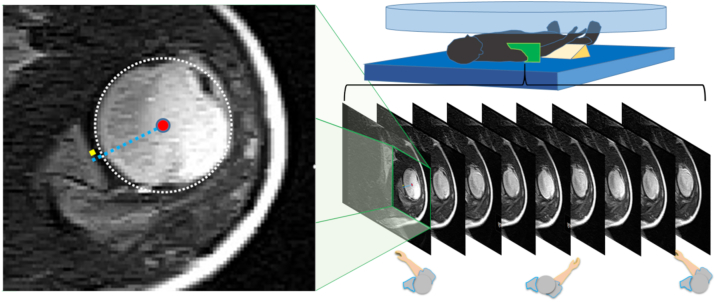
MRI scanning sequence and data analysis. Participant position during active shoulder rotation in the MRI scanner (*Right* figure) and measurement of the humeral head position (*Left* figure). The center of the humeral head was calculated using the least-squares method based on the coordinates traced on the articular surface. The *light blue line* projects the center point of the humeral head vertically onto the glenoid fossa. The *yellow dots* indicate the midpoint of the glenoid. Humeral head deviation was measured as the distance between the *light blue line* and the *yellow dot* on the glenoid fossa. *MRI*, magnetic resonance imaging.

All participants underwent glenohumeral instability testing (load and shift, anterior drawer, posterior drawer, apprehension, and sulcus tests) conducted by a certified physical therapist specialized in musculoskeletal disorders prior to cine MRI scanning. During scanning, the participants lay supine and performed shoulder rotation with their arms positioned at the sides of their bodies (Fig. 1). Active internal and external shoulder rotations were performed at a rate of 15 cycles per minute, guided by a digital metronome, as previously described.^8^

Cine MRI was performed using a 0.4 Tesla open MRI system (Aoerti Eterna, Hitachi Medical Corporation, Japan). The scanning parameters for the modified gradient echo sequence were as follows: repetition time/echo time = 4.4 ms/2.2 ms, flip angle = 90°, slice thickness = 1.7 mm, bandwidth = 160 kHz, field of view = 32 × 32 cm, and matrix = 256 × 256 pixels. Axial slices were acquired at a rate of two images per second to capture intra-articular motion during active shoulder rotation.^8^

### Sample size estimation

The sample size was calculated using R software (version 4.5.1; R Foundation for Statistical Computing, Vienna, Austria). To our knowledge, no previous studies have quantitatively assessed dynamic glenohumeral head deviation in MDI using cine MRI. Therefore, the calculation was based on a prior study^11^ that was the only available study using a comparable methodology. That study reported posterior humeral head displacement of 2.44 ± 0.56 mm in normal shoulders and 4.22 ± 0.23 mm in post-Remplissage with Bankart repair shoulders during active external rotation in the abducted position, yielding an estimated effect size (Cohen's d) of 4.26. While this study examined a different pathology, it represented the most relevant reference for estimating effect size in dynamic humeral head translation. A two-sided t-test with α = 0.05 and power = 0.80 indicated that a minimum of two shoulders per group would be required to detect this difference. To account for potential variability in the MDI and ensure group representation, the target sample size was set at least seven shoulders per group.

### Data analysis

All measurements were performed independently by a single observer who was blinded to the clinical diagnosis at the time of measurement. MATLAB (version R2023b; MathWorks Inc., Natick, MA, USA) was used to analyze the cine MRI data. The center of the humeral head was determined using a least-squares circle-fitting algorithm, and the direction of glenohumeral rotation was monitored frame by frame.^8^ The center of the glenoid fossa was defined as the midpoint between the anterior and posterior rims. The humeral head position was calculated as the perpendicular projection of the center of the humeral head onto the glenoid plane.

Humeral head deviation was defined as the distance between the sequential positions of the humeral head center.^8^ The following outcome measures were derived:

• Maximum humeral head deviation: The greatest cumulative translation in a single direction during active rotation.
• Amplitude of humeral head deviation: distance between the maximum anterior and posterior deviations of the humeral head.
• Humeral head deviation rate: The maximum humeral head deviation was expressed as a percentage of the glenoid width.
• Amplitude of deviation rate: The combined anterior and posterior deviation rates.
• Maximum deviation velocity: The peak instantaneous translation speed of the humeral head, calculated as the maximum deviation between two consecutive MRI frames divided by the frame interval (0.5 s).

The reliability and validity of these measurement methods was confirmed in previous study.^8^

### Statistical analysis

Statistical analyses were performed using R software (version 4.5.1; R Foundation for Statistical Computing, Vienna, Austria). Welch's t-test was used to compare the MDI and control groups for continuous variables including age, glenoid width, and all kinematic parameters. Effect sizes (Cohen's d) were calculated to quantify the magnitude of the differences between groups. Categorical variables, such as sex and dominance, were compared using the chi-square test. Statistical significance was set at *P* < .05.

### Ethics

This study was approved by the Ethics Committee of Nagoya University (approval no. 14-504). All participants provided written informed consent after receiving comprehensive verbal and written explanations of the study procedures, including the MRI scanning protocols, potential risks, and data usage. Patient data were deidentified and anonymized prior to analysis to ensure confidentiality of the data. This study was conducted in accordance with the principles of the Declaration of Helsinki.

## Results

### Participant characteristics

The characteristics of the participants are shown in Table I. No significant differences were observed between the MDI and the control groups in terms of age, sex, limb dominance, humeral head radius, or glenoid width (*P* > .05). However, the range of shoulder rotation with the arm at the side was significantly greater in the MDI group than that in the control group (93.5° ± 11.0° vs. 77.3° ± 18.8°, *P* = .010). Among the eight MDI shoulders, anterior instability was present in six, posterior instability in five, and inferior instability in all cases. One participant reported apprehension during both manual examination and cine MRI testing.

**Table I:** Participant profiles (mean ± standard deviation).

Characteristic	MDI group (n = 8 shoulders)	Control group (n = 20 shoulders)	*P* value
Age (yr)	29.8 ± 8.4	27.8 ± 6.1	.694
Gender (male/female)	2/2	8/2	.263
Dominant hand (right/left)	4/0	10/0	-
Radius of humeral head (mm)	19.8 ± 1.2	20.5 ± 1.4	.168
Glenoid width (mm)	22.1 ± 1.9	23.9 ± 2.7	.07
Rotation range (degree)	93.5 ± 11.0	77.3 ± 18.8	.010
Anterior instability (shoulders)	6	0	-
Posterior instability (shoulders)	5	0	-
Inferior instability (shoulders)	8	0	-

*-*, not applicable; *MDI*, multidirectional glenohumeral instability; *SD*, standard deviation.

Demographic and anatomical characteristics of the MDI and control groups.

The radius of the humeral head was calculated by fitting a reference circle using the least-squares method (as shown in Fig. 1). Values are presented as mean ± SD or counts. *P* < .05 indicates statistical significance.

### Humeral head deviation

**Figure 2:**
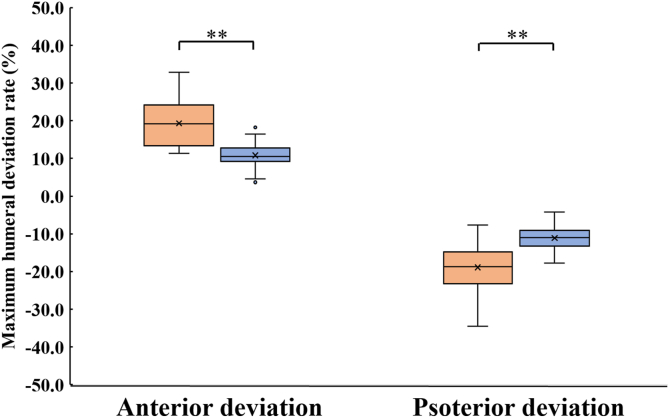
Maximum humeral head deviation rate. Box plots illustrating the maximum humeral head deviation rate in the MDI (*red*) and control (*blue*) groups. A positive value indicates anterior deviation, and a negative value indicates posterior deviation. The ±50% thresholds represent the anterior and posterior glenoid edges, respectively. ∗∗: *P* < .01; ∗: *P* < .05. *MDI*, multidirectional glenohumeral instability.

The MDI group exhibited significantly greater humeral head deviation during active shoulder rotation than the control group (Fig. 2). Both maximum anterior and posterior deviations were significantly larger in the MDI group compared to the control group (*P* = .008 and *P* = .009, respectively). The maximum anterior deviation was 4.3 ± 1.4 mm in the MDI group and 2.6 ± 0.7 mm in the control group, with a mean difference of 1.7 mm (95% confidence interval [95% CI]: 0.6-2.8 mm, Cohen's d = 1.92). The maximum posterior deviation was 4.2 ± 1.7 mm in the MDI group and 2.6 ± 0.7 mm in the control group, with a mean difference of 1.6 mm (95% CI: 0.2-3.1 mm, Cohen's d = 1.51).

### Amplitude of humeral head deviation

**Figure 3:**
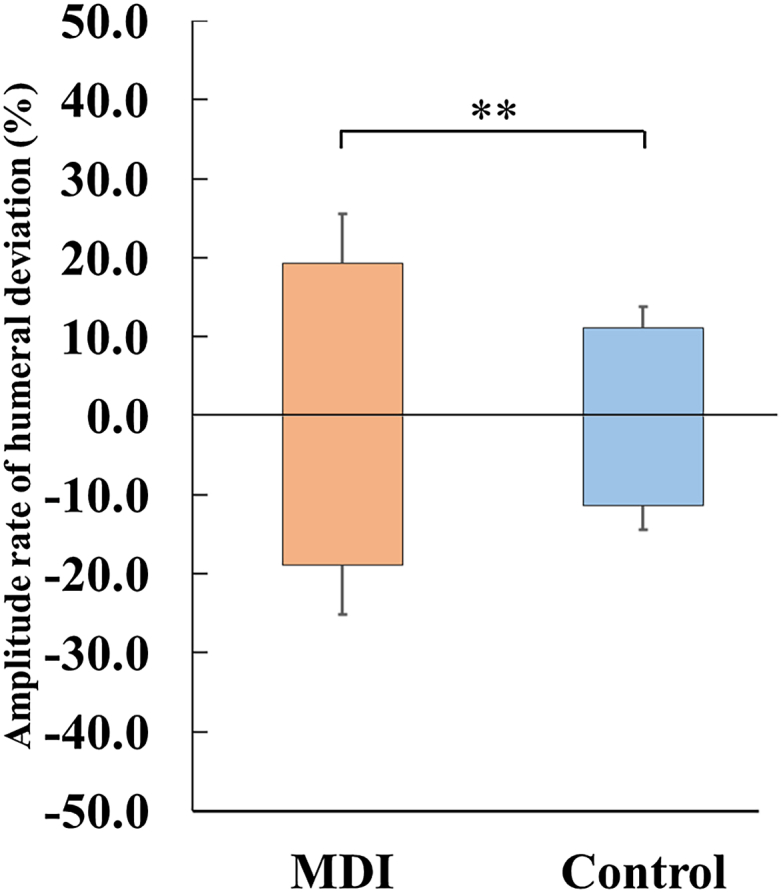
Amplitude of the humeral head deviation rate. The shaded areas indicate the amplitude of humeral head deviation in the MDI (*red*) and control (*blue*) groups. Positive and negative values indicate anterior and posterior deviation, respectively, with ±50% corresponding to the anterior and posterior glenoid margins. ∗∗: *P* < .01. *MDI*, multidirectional glenohumeral instability.

The combined amplitude of humeral head deviation (anterior plus posterior displacement) was significantly wider in the MDI group (9.5 ± 2.4 mm) than in the control group (5.9 ± 1.1 mm), with a mean difference of 3.6 mm (95% CI: 1.6-5.6 mm, Cohen's d = 2.32, *P* < .001). When normalized to glenoid width, the mean deviation amplitude exceeded 35% in the MDI group, surpassing established clinical thresholds for pathological translation.^1^ The anterior and posterior deviation rates were 22.1 ± 6.1% and 21.1 ± 6.8% in the MDI group, compared with 12.1 ± 2.9% and 12.3 ± 3.1% in control groups (*P* = .002 for both directions) (Fig. 3).

### Humeral head deviation velocity

**Figure 4:**
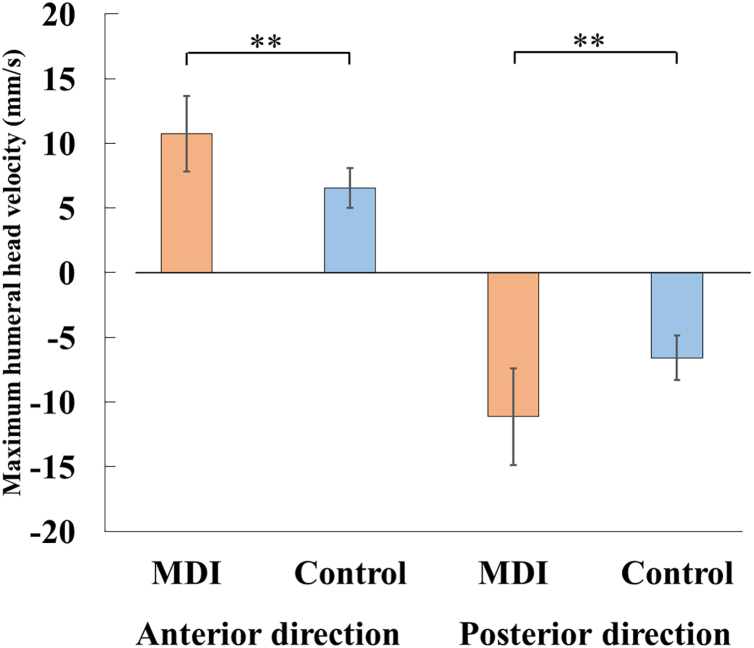
Maximum humeral head velocity. Bar graphs showing the maximum deviation velocity of the humeral head during external (anterior) and internal (posterior) rotation in the MDI (*red*) and control (*blue*) groups. Positive and negative values indicate anterior and posterior velocities, respectively. ∗∗: *P* < .01. *MDI*, multidirectional glenohumeral instability.

The maximum velocity of humeral head deviation was also significantly higher in the MDI group (Fig. 4). During external rotation (anterior direction), the velocity was 10.5 ± 3.2 mm/s in the MDI group and 6.2 ± 1.6 mm/s in the control group, with a mean difference of 4.3 mm/s (95% CI: 1.6-7.0 mm/s, Cohen's d = 2.00, *P* = .006). During internal rotation (posterior direction), the velocity was 11.4 ± 2.8 mm/s in the MDI group and 6.4 ± 1.7 mm/s in the control group, with a mean difference of 4.9 mm/s (95% CI: 2.5-7.4 mm/s, Cohen's d = 2.36, *P* = .001).

These findings demonstrate that shoulders with MDI exhibit a significantly greater magnitude and velocity of humeral head motion during active rotation than healthy controls.

## Discussion

This study confirmed all three hypotheses by quantitatively demonstrating that patients with MDI exhibited significantly wider amplitude of humeral head deviation as a result of greater anterior and posterior humeral head deviations, and faster deviation velocity during active shoulder rotation than healthy controls. These findings provide objective validation for the clinical concept of dynamic glenohumeral instability.

These measurements provide mechanistic insights into MDI pathophysiology by translating tactile clinical impressions into objective evidence of abnormal humeral head motion patterns.

The increased magnitude of deviation provides objective evidence to support the clinical impression that humeral head translation appears greater than expected during manual palpation. The mean deviation amplitude of 8.4 mm (>35% of the glenoid width) exceeded the 25% threshold commonly used to define pathological translation in load-and-shift testing.^1^ This objective threshold supports the clinical interpretation of pathological instability. Importantly, cine MRI may enhance patient understanding through visual confirmation of instability and improve diagnostic precision when manual testing yields equivocal results.

The increased posterior deviation observed in the MDI group may reflect a combination of capsular laxity and impaired dynamic stabilization. Previous electromyographic studies have documented altered rotator cuff activation timing in patients with MDI, supporting this interpretation.^5^^,^^9^^,^^12^ The wide range of deviation amplitudes (23.1% to 60.0% of glenoid width) suggests that passive structures alone cannot explain the observed instability, even during the controlled motion. The 4.3-5.0 mm/s faster deviation velocity may reflect a failure of dynamic control mechanisms to maintain humeral head centering during rotation. This finding provides quantitative evidence supporting the clinical perception of sudden humeral head translation during manual examination.

To minimize intersubject variability in movement speed, all participants performed shoulder rotation at a standardized rate of 15 cycles/min, guided by a digital metronome. However, interindividual differences in muscle strength and neuromuscular control may have influenced the velocity measurements. Despite this potential variability, the large effect sizes observed suggest that the differences in deviation velocity between the MDI and control groups reflect underlying dynamic instability rather than general movement variability.

The supine testing position was necessary due to imaging constraints, as simultaneous visualization of humeral head and glenoid was not possible in functional positions like abduction and external rotation (ABER). While less provocative than ABER positioning, this approach enables a consistent assessment of intra-articular motion. Traditional imaging modalities provide structural information but cannot capture real-time joint kinematics. Cine MRI enables noninvasive assessment without probe-induced mechanical restriction, unlike ultrasound or radiation exposure risk, unlike radiography.

This study had two limitations. First, the sample size was relatively small (n = 8 MDI shoulders), and the absence of a clinical correlation limited the generalizability of the findings. However, the observed effect sizes were substantial (Cohen's d = 2.35 for anterior deviation, d = 2.30 for posterior deviation), considerably exceeding the conventional threshold for large effects (d = 0.8). These large effect sizes retrospectively confirmed the adequate statistical power to detect clinically meaningful differences, despite the limited sample size. While this established reliability supports the reproducibility of the measurements in this study, an interobserver reliability assessment would strengthen confidence in the generalizability of this measurement approach for future studies. Nevertheless, validation in larger, multicenter cohorts with clinical correlation is essential to establish normative thresholds for clinical application, assess the full spectrum of MDI presentations, and confirm the diagnostic utility of this method. Second, the supine positioning adopted for imaging may not accurately represent functional instability patterns. Future studies should investigate how cine MRI might contribute to treatment decision-making and objective monitoring of therapeutic progress. In addition, research should explore instability patterns under more rapid, antigravity conditions and in functional joint positions such as ABER.

These findings suggest potential clinical implications for MDI management. The quantitative measurements obtained in this study may contribute to objective assessment when manual examination findings are equivocal, although further validation is required to establish the diagnostic thresholds. Furthermore, cine MRI may have potential utility in treatment planning by providing baseline measurements and enabling objective monitoring of rehabilitation progress, as successful neuromuscular training would theoretically reduce both deviation amplitude and velocity. The visualization capability may also enhance patient education by demonstrating the mechanical basis of their symptoms. However, larger prospective studies with clinical correlations are necessary to confirm these potential applications and establish the role of cine MRI in clinical decision-making.

## Conclusion

This study tested the hypothesis that patients with multidirectional instability (MDI) would demonstrate greater humeral head deviation and faster deviation velocity than healthy controls during active shoulder rotation. The results confirmed this hypothesis, with MDI patients showing significantly greater deviation in both directions and higher velocities (Cohen's d > 1.5 for all comparisons). Cine MRI successfully quantified dynamic glenohumeral instability, demonstrating its potential as an objective assessment tool for MDI.

## Acknowledgment

The authors would like to express their deepest gratitude to Dr. Katsuya Nobuhara, who passed away on March 13, 2022. He organized patient recruitment, modified the scan schedule using a magnetic resonance imaging device, and analyzed the data. The authors would also like to express their gratitude to Shigetoshi Morioka, Koji Tomita, and Mutsuki Takeuchi, radiologists at Nobuhara Hospital, who captured magnetic resonace images of participants' shoulders.

## Disclaimers:

Funding: No funding was disclosed by the authors.

Conflicts of interest: The authors, their immediate families, and any research foundation with which they are affiliated have not received any financial payments or other benefits from any commercial entity related to the subject of this article.
